# Diagnosis and Treatment of Small Bowel Cancers Using Radioactive Gold Nanoparticles and Wireless Fluorescence Capsule Endoscopy

**Published:** 2016-03-01

**Authors:** M. Alizadeh, V. Qaradaghi

**Affiliations:** 1 Department of Bioengineering, Temple University, Philadelphia, USA; 2 Department of Electrical Engineering, University of Texas at Dallas, Dallas, USA

**Keywords:** Radioactive gold nanoparticle, Wireless fluorescence capsule endoscopy, Small intestine, Polyethylene glycol, Dose, activity

## Abstract

**Background:**

Therapeutic and diagnosis properties of radioactive gold nanoparticle (198-AuNPs) cause them to be suitable for detection and treatment of tumors.

**Objective:**

Electrical and optical properties of PEG-198AuNPs were examined in this paper. Polyethylene Glycol (PEG)-198 AuNPs can be used for treatment and diagnosis of small intestine tumors.

**Methods:**

Wireless fluorescence capsule endoscopy will be able to detect emission lights of triggered Au by external light. First, the output electrical field was calculated by DDSCAT software. Secondly, tumor and distribution of PEG-198 gold nanoparticles were modeled using Monte Carlo simulation and finally dose delivered throughout a solid tumor when the PEG-198 gold nanoparticles linked to each cell was calculated.

**Results:**

Polyethylene Glycol functionalized gold nanoparticles (AuNPs) possess optimized sizes (30 nm core diameter and 70 nm hydrodynamic diameters) to target individual tumor cells. Surface distribution to receive doses of up to 50Gy was simulated.  Activities and absorbed doses by the tumors with 0.25cm and 0.5cm radius were 187.9mCi and 300mCi and 72 and 118 Gy,respectively.

**Conclusion:**

Therapeutic and diagnosis properties of 198-AuNPs show that it can be used for treatment and detection of small bowel tumors in early stage of growing.

## Introduction

**Figure 1:**
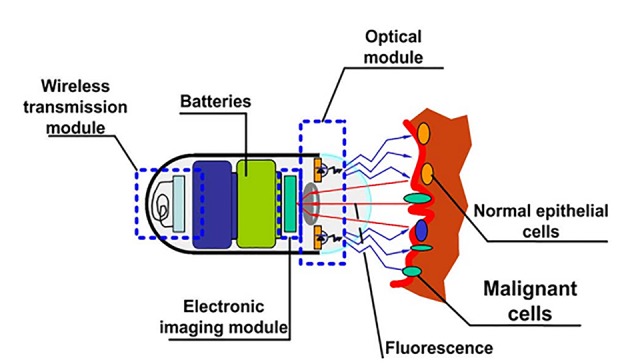
Conceptual design of the wireless fluorescence imaging system for noninvasive clinical diagnosis [14].

Small bowel cancer starts when cells in the lining of the small bowel (also called the small intestine) change and grow uncontrollably, forming a mass called a tumor. A tumor can be noncancerous or malignant. These changes can take a long time to develop. Both genetic and environmental factors can cause such changes, although the specific causes of small bowel cancers are generally not well understood. Nanotechnology has the potential to provide a paradigm shift in the way diagnostic and  therapeutic  drugs are  delivered  to achieve  target  specificity and  inversed retention  for considerable  improvement  in the  overall  treatment of  the  small intestine  and various inoperable tumors[1]. Gold nanoparticles have unique optical and electronic properties that bulk material does not present[2]. In the ancient times, these nanoparticles had been used to make windows glasses red in color. Gold nanoparticles (AuNPs) can be used to guide, enhance, emit, and modify the optical ﬁelds[3], and also have been used recently for biosensing and in drug delivery systems[4]. Gold  nanoparticles (AuNPs)  are  currently being  investigated  for their  potential  uses in targeted cancer detection and treatment. The small size and configuration of NPs also allow them to pass through biological membranes into cells, where they can act as targeted drug delivery systems for malignant cells while sparing healthy neighboring cells[5]. Recent work by Kannan et. al investigated gum Arabic labeled radioactive AuNPs that localize in liver. This study combine the therapeutic property of radioactive gold 198-Au (β_max_=0.96MeV; half-life of 2.7 days) and target specific biomolecule to form a powerful radiopharmaceutical fortargeted drug delivery. Also, Blogh et. al has made ground breaking contributions toward the fabrication of poly radioactive gold dendrimer composite nano-devices of sizes between 10nm and 29nm demonstrated their utility in targeted radiopharmaceutical dose delivery to tumors[6], and Laura and co-workers showed that AuNP-T cell group exhibited significant higher gold delivery to the lung, liver, and bone, while the PEG-AuNP demonstrated higher gold level within the small bowel[7]. Results show that for beta- emitting nanoparticles, a set of data (covering fraction, biological half-life,  and nanoparticles  radius) can  be found  within acceptable ranges (these of classical radio immunotherapy). These source (with E_max_~ few MeV) can be used for the treatment of tumor with a maximum diameter of 1 cm. Thus low- energy X- rays (E_mean_<25keV) can be used to extend the range of tumor diameter to 4-5cm but required very tight biological vector characteristics[8]. We can use the optical properties of gold nanoparticles for cancer imaging. One of the devices for cancer imaging is wireless capsule endoscopy[9-13]. Fluorescence-imaging-based wireless endoscopy can be used for screening and diagnostic application in the GI tract[14]. Figure 1 shows the parts of a wireless fluorescence capsule endoscopy system. Wireless Capsule Endoscopy (WCE) is a relatively new technology (FDA approved in 2002) allowing doctors to view most of the small intestine. Previous endoscopic imaging modalities such as colonoscopy, upper gastrointestinal endoscopy, push enteroscopy and intraoperative enteroscopy could be used to visualize up to the stomach, duodenum, colon and terminal ileum, but most of the small intestine could not be viewed without surgery. Recently, there has been a great deal of progress in the development of wireless capsule endoscopy for taking video images of digestive organs[9-13].

## Material and Methods

### Gold Nanoparticle Effects in Plasmon Resonance

When a small spherical gold nanoparticle is irradiated by the light, the electric field oscillation causes the conduction electrons oscillate coherently[15].The skin depth of bulk gold for visible light is 35 nm[16]. Therefore, the incident light can go through the AuNPs with the diameters of 10 to 20 nm. Thus the generated electromagnetic wave leads to a particle polarization. Due to the illumination, the electrons are shifted to one side of the particle and the atomic cores remain as a positive charge on the other side. The collective oscillation of electrons is called the dipole Plasmon resonance of particle. Gustav Mie has characterized the electrical and optical properties of nanoparticles[17].

Mie developed a theory that describes the scattering of incident light by the spherical particles. He presented a solution to the Maxwell’s equations that describes the extinction spectra (scattering plus absorption) of spherical particles with arbitrary sizes. There are many published papers regarding AuNP’s characteristics and surface Plasmon. These papers, e.g.[15] explain more details of Plasmon resonance AuNPs. It should be noted that we do not describe the details in this paper and the reader can refer to the literature for more information. If we take into account only the double precision for a simple AuNP, Eq 1 could be a suitable relation for the explanation of the output electric field. This equation shows the output generated electric field *E_out _*due to the incident electric field applied to the nanoparticles[15].

$$E_{out}=E_{0}{\hat{a}}_{x}-{a.E}_{0}\left[\frac{\hat{x}}{r^{3}}-\frac{3x}{r^{5}}({x\hat{a}}_{x}+{{y\hat{a}}_{y}+z\hat{a}}_{z})\right]\;\left(1\right)$$

Where *E*_0_ is the value of incident electric field which we assume is applied in *x̂* direction. Also *â*_x_ , *â*_y_ and *â*_z_ are the traditional unit vectors in *x̂*, *ŷ* and *ẑ* directions. *α* is the polarizability and can be expressed as:

$$a=\frac{\varepsilon_{i}-\varepsilon_{0}}{\varepsilon_{i}+{2\varepsilon }_{0}}a^{3}\;\left(2\right)$$

where *ε*_0_ and *ε_i_* are the vacuum permittivity and the relative permittivity of AuNp, respectively, and a is the radius of AuNP. It should be noted that, *ε_i_* is strongly dependent on the wavelength of incident electric field.

### Tumor Models

The MCNP software is capable of simulating the transport of photons and electrons in matter over a broad energy range.  The input file of this code is composed of three types of data, those corresponding to the geometry of the problem (composition, form, and density of the tumor and surrounding healthy tissues), the radioactive sources (nature, emission, spectra, and positions inside and near the tumor), and the nature of the desired result (i.e., the deposited energy)[18].There are three distinct stages (avascular, vascular and metastatic) to cancer development and then researchers often concentrate their efforts on answering specific question on each of these stages[19]. In this paper we consider the avascular model.

**Figure 2:**
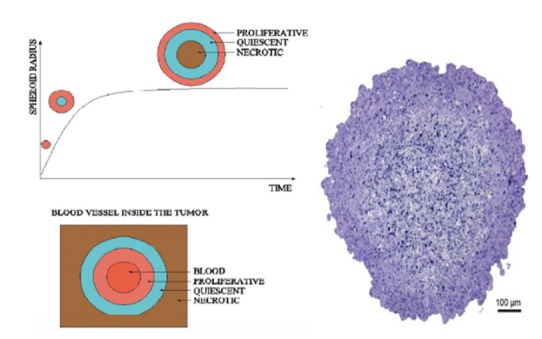
Left : Schematic illustration showing tumor spheroid growth, Right : 1 micron of spheroid section showing the proliferative rim and necrotic core [20].

Avascular tumor growth is much simpler to model mathematically and yet contains many of the phenomena that we need to address in a general model of vascular tumor growth. Thus we see the modeling of avascular tumors as a first step toward building models for fully vascularized tumors. In addition, there are some questions concerning avascular tumors which may be interesting in their own right, including the recent controversial hypothesis that all humans have small dormant avascular tumors in their bodies[18, 20]. There are also parallels between avascular tumor growth and the growth of a tumor tissue in the micro region supported by a single blood vessel inside a vascular tumor, as illustrated in Figure 2, where the different region of both avascular and vascular tumors are shown. Thus, avascular tumor modeling can be of use when making predictions and designing experiments on vascular and metastatic tumors, which are much more time consuming and difficult as they have to be performed in vivo[20].

### Range- Energy Relationship for Beta Particles of 198- Gold Nanoparticles in Tissue

The quantitative relationship between beta energy and range is given by the following experimentally determined empirical equation:

R = 0.542E - 0.133 E ≥ 0.8Mev              (3)

where R=range(r/cm^2^) and E=maximum beta energy (MeV). Here E=0.96 MeV then R=0.38732 (g/cm^2^) linear thickness is found from:

$$t_{d}\left(\frac{g}{{cm}^{2}}\right)=p\left(\frac{g}{{cm}^{3}}\right)*t_{1}(cm)\;\left(4\right)$$

$$t_{1}(cm)=\frac{t_{d}\left(\frac{g}{{cm}^{2}}\right)}{P_{small\;intestine}\left(\frac{g}{{cm}^{3}}\right)}=\frac{R\left(\frac{g}{{cm}^{2}}\right)}{P_{small\;intestine}\left(\frac{g}{{cm}^{3}}\right)}=\frac{0.38732\left(\frac{g}{{cm}^{2}}\right)}{1.05\left(\frac{g}{{cm}^{3}}\right)}\approx 0.37(cm)$$

where *t_d_* is the density thickness, *ρ* is the density, and *t_l_*is the linear thickness[21, 22].

### Calculation of Average Number of Gold Atoms per Nanoparticles

The average number of gold atoms per nanoparticles maybe calculated from high resolution TEM analysis. The average number of gold atoms (N) for each type of nano sphere was calculated by Eq 5. Where *ρ* is the density of gold (19.3 g/cm^3^) and M stands for atomic weight of gold (197 g/mol)[23].

$$N=\frac{\pi }{6}\frac{{pD}^{3}}{M}={30.89602D}^{3}\;\left(5\right)$$

### Dose Calculation

The use of the tally F8 (MeV/g/nps) proposed by Nuttens and co-workers to determine the distribution of dose according to the distance from the tumor center is no longer valid for these new vascularized models. Indeed, the tally F8 calculates the energy deposition in a cell, which is defined by a single density and simple geometry[18]. In order to estimate the dose delivered to the tumor and the healthy tissues, the activity (in mCi) at the spherical surface has to be deduced from:

$$A=\frac{\lambda_{phys}.n_{a}.n_{bv}}{3.7*10^{7}}\;\left(6\right)$$

where *λ_phys_* is radioactive decay constant of the emitter (in*h*^-1^). The number *n_bv_* of biological vector that can be bound at the tumor surface is obtained by multiplying the solid tumor surface by the covering fraction. The number *n_a_* of radioactive atoms per nanoparticles is *n_a_*=* c_r_*×* ρ_molecule_*×(4*πr*^3^/3), where *c_r_* is the number of radioactive atoms per molecule, *ρ_molecule_* is the number of molecules per cm^3^, and *r* is the nanoparticles radius. The deposited dose (in Gy) is obtained from the expression:

$$D=\frac{XTpA}{\lambda_{eff}}\;\left(7\right)$$

where *T* is the tally value in MeV/g per emitted particles; P (in *dis*^-1^) is the average number of particles (electron of photon) emitted per disintegration; *A* is the activity (mCi); and *λ_eff_*=* λ_bio_*+* λ_phy_s *(in*h*^-1^), *x*=21.34 is a constant to convert dose rate from mCi MeV/g/dis to Gy/h [8].

## Results

*Electrical properties of AuNPs*

**Figure 3:**
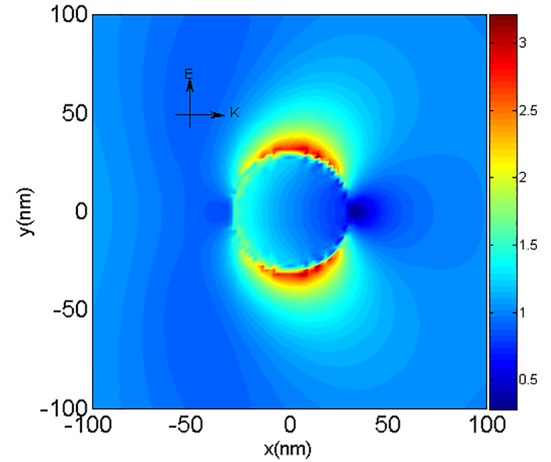
The generated output electric field for a singleAuNp with the radius of 30 nm and the wavelength of 250 nm.

In this paper, the plasmon resonance excitation due to the gold nanoparticles (AuNPs) was described. For the calculation of electric field inside and outside the nanoparticle, DDSCAT software which uses a method known as the Discrete Dipole Approximation (DDA) was used[24] The DDA algorithm is a numerical method in which the object of interest is represented as a cubic lattice of N polarizable points. The electric field for one AuNP and the interaction effects between particles (particle-particle interactions) have been studied in this paper. Also the dependence of output signal fluorescence to the particle size and polarity was studied too. Figure 3 shows the generated electric field for a single AuNp with the radius of 30 nm and the wavelength of 250nm. For the calculation of generated electric field, DDSCAT 7.1 (Discrete Dipole Approximation Code) software was used[24]. This software calculates the scattering and absorption of electromagnetic field for a target with arbitrary geometry and complex refractive index, using the DDA method. Also this software calculates the electric and magnetic fields for nanoparticles.

As shown in the figure 3, intensity of electric field near the NPs is higher than far distances. Therefore, fluorescence emitted light intensities that triggered by this electric field have a large amount near the NPs. Emitted lights by NPs show the place of tumors if NPs localize in tumor, accurately.

*Dose calculation*

**Figure 4:**
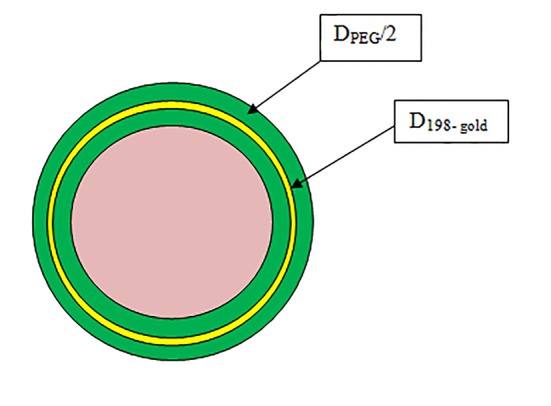
Surface distribution of PEG-198AuNP around a tumor

**Figure 5:**
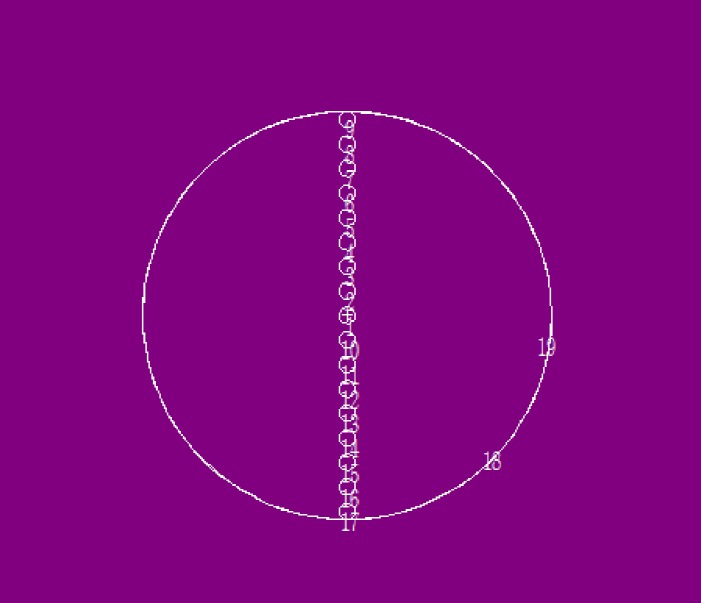
Monte Carlo modeling with micro cells to calculate absorbed dose

PEG-radioactive gold nanoparticles (AuNPs) possess optimized sizes (30 nm core diameter and70 nmPEG diameter) to target individual tumor cells and penetrate through tumor vasculature and pores, and we consider nanoparticle dimensions and biological vector characteristics in order to reach 50 Gy dose inside solid tumors[25].There are different models that suggest for NPs distribution in tumors. The radioactivity distribution throughout the tumor volume can be uniform, linear or exponential and surface. Stage of tumor growing is important factor to select these models. In surface distribution, maximum dose will be delivered near the surface and minimum dose will be delivered in the center of tumor. This model is not accurate, especially in large tumors. But this model is easy to conception of NPs distribution and simulation. Activity and absorb dose considering surface distribution of NPs as shown in Figure 4 and. For the 1mg/milt uptake of PEG-198AuNPs in the small intestine tumors with 0.25 cm and 0.5cm radius were 185.6mci, 212.4mci, 71.5 and 89.9 Gy, respectively. Due to the short physical half-life of this radioelement (* T_phys_*=2.67 days), only 60% was consider to be reached in the tumor. In order to calculate dose near the surface, micro cells with 0.2mm radius and 0.1 mm distance from each other as shown in Figure 5 was considered.

As shown in table 1 and table 2, minimum and maximum absorbed dose will be deposited at the center and near the surface of tumors, respectively. It’s easy to concept of these results by range energy relationship for beta particles from equation 3. Minimum absorbed dose will be received in the center of tumors. For large tumors (i.e. larger than 0.37 cm) absorbed doses are nearly zeros near the tumor center.

**Table 1:** Simulated dose in a tumor with 0.25cm radius.

**Distance from surface (mm)**	**Dose (Gy)**
0.3	71.5
0.8	56.2
1.3	42.3
1.8	21.5
2.3	10.75

**Table 2:** Simulated dose in a tumor with 0.5cm radius.

**Distance from surface (mm)**	**Dose (Gy)**
0.3	89.9
0.8	59.5
1.3	35.1
1.8	18.1
2.3	8.9
2.8	1.1
3.3	0.56
3.8	0.1
4.3	0
4.8	0

## Discussion

In previous researches, it has shown that particle size plays a key role in the final bio-distribution and blood clearance of stealth particles. It is reported that, molecules that have a molecular weight less than 5000, or even higher for dense polymers such as dendrimers, can be removed from the body via the renal system. For large molecules and particles that can not be removed by the renal system, research has shown that particles with hydrodynamic radii of over 200 nm typically exhibit a more rapid rate of clearance than particles with radii under 200 nm, regardless of whether they are PEGylated or not. 

## Conclusion

A novel system was proposed for imaging and treatment of small intestine tumors in this paper. Therapeutic and diagnosis properties of 198-AuNPs for treatment and detection of small bowel tumors was used in this study. The optical properties of AuNPs for were considered as a way of detection of tumors by wireless fluorescence imaging system. So, small intestine can be detected in the early stage of tumor growing. Fluorescence lights can be detected by wireless fluorescence capsule if AuNPs be triggered by external light. Another advantage of 198AuNPs is that these AuNPs deliver high dose rate to tumor cells with minimum negative effects on normal cells. 
